# Acute effects of differential learning on football kicking performance and in countermovement jump

**DOI:** 10.1371/journal.pone.0224280

**Published:** 2019-10-23

**Authors:** Alex Gaspar, Sara Santos, Diogo Coutinho, Bruno Gonçalves, Jaime Sampaio, Nuno Leite

**Affiliations:** 1 Faculty of Human Sciences, Otto-von-Guericke Universitat, Magdeburg, Germany; 2 University institute of Maia, ISMAI, Maia, Portugal; 3 Research Centre in Sports Sciences, Health Sciences and Human Development, CIDESD, CreativeLab Research Community, Vila Real, Portugal; 4 Department of Sports Sciences, Exercise and Health, University of Trás-os-Montes and Alto Douro, Vila Real, Portugal; Universidade Federal de Mato Grosso do Sul, BRAZIL

## Abstract

The aim of this study was to identify the acute effects of a differential-learning training program on football kicking performance and countermovement jump. Twenty youth Portuguese under-15 football players participated in this study. All players were exposed to two training approaches: i) traditional, in which the players performed a total of 36 kicks in a blocked and repetitive approach; and ii) differential learning, which consisted in the 36 kicks using differential variations in each kick. Football kicking impact and velocity were assessed using a Stalker radar gun, while the kicking accuracy was assessed by aggregating the total number of points achieved during 12 kicks into a goal, which was divided into quantifiable scoring zones. Lastly, leg power was measured using a countermovement jump. Measurements were performed at baseline, post-intervention, and following a 35-minute training match. The comparisons between the baseline and post-test revealed that the differential learning approach promoted a possibly ~5% increase in the countermovement jump (small effects) and a likely ~3% increase in the average velocity (small effects) when compared with the traditional training approach. From the accuracy perspective, there was a moderate decrease from the baseline to the post-test and post-match in accurate kicks into zone 1 (centre of the goal) and a moderate decrease from the baseline to the post-match in accurate kicks into zone 5 (lateral zones at short height) in the differential intervention. In turn, a small increase in the accurate kicks into zones 4 and 6 (lateral zones of the goal and nearest to the bar, respectively) was found from the baseline to the post-match in the differential intervention. Overall, the differential learning intervention was more beneficial than a traditional training protocol with respect to acute improvements in countermovement jump performance, football kicking velocity and higher scoring zones kicking accuracy.

## Introduction

Performance in association football can be characterized by its integration of physical [1], tactical [2], and technical mastery [3]. Accordingly, a considerable effort has been made to optimize these three dimensions, in an attempt to enhance the performance of individuals and teams. While success results from complex interactions of these variables, the technical actions that can be determinants of team success have received considerable attention [2, 4]. Performance indicators including total kicks, kicks on target, ball possession, passing, corner kicks, have all been the subject of investigation in regards to differentiating successful teams in association football. According to Castellano and collaborators [4], one of the offensive performance indicators that best differentiated between successful and unsuccessful teams included total kicks performed and the number of kicks on target. Due to the proven importance of kicking actions related to success in football, mastery of this specific technical parameter has been of significant scientific interest.

Kicking is considered as one of the most fundamental actions performed in a football game [5] and is produced by the coordination of body segments with the intention of striking the ball with velocity and precision towards a target (the goal) [6]. Previous research examining kicking performance has been done in several ways, largely in the form of biomechanical analyses examining kinetic and kinematic differences that arise between participants and how these differences ultimately manifest themselves in kicking success [7]. Although the meaning of kicking success is open to interpretation, the speed exhibited by the ball has become the main biomechanical indicator when performing a football kick task [7, 8]. Despite the emphasis on speed, kicking mastery must also include an element of accuracy, as the dynamic nature of football requires players to successfully manipulate ball speed and trajectory when directing a kick toward the goal defended by an opponent’s goalkeeper [9]. Another avenue of kicking performance research is the implementation of interventions aimed at enhancing its performance. Resistance training [10], plyometric training [11], sprint training [12], proprioceptive neuromuscular facilitation [13], and other methods have all been employed as a supplement to regularly scheduled training of football players, in an effort to elicit changes in kicking performance. It is important to note that while many of these interventions were performed with the overall aim of enhancing kicking performance, increasing lower limb strength was the mechanism selected to achieve a greater performance. The justification behind this methodology appears to be that increasing lower limb power has transferability to football-specific actions, and that ball speed would be positively affected by any changes that arise in strength/explosiveness of the lower limbs [14].

Additionally, many of the interventions applied to improve kicking performance favoured repetitive movements, which has been seen as the basis for creating changes in association football technical actions. While these investigations have provided useful information related to kicking performance, other types of training methods must be considered in order to establish a holistic understanding of football kicking training. The concept of incorporating variability into practice was largely pioneered by Schmidt [15], who later popularized the idea of introducing movement variability to the training context as a way to facilitate motor learning [16]. Therefore, variability is seen as an essential component to training and learning, and this premise is reinforced in the differential learning approach [17]. Differential learning is characterized by challenging the participant to perform a variety of exercises, without repetition, that mimic some of the many environmental conditions in which they will have to reproduce the movement in [18–21]. The variations expressed in differential learning training include changes to any combination of the following features: joint manipulation, movement geometry, movement speed, equipment variation, and environmental variation [22]. These fluctuations create a necessity for adaptation and force players to create unusual but appropriate movement responses. Accordingly, recent studies highlight differential learning as a promising approach to nurture adaptive behaviour in youth football players [20, 23].

Differential learning training has been applied in a number of sports and has illustrated favourable results in speed skating [18], badminton [24], basketball [23, 25], hockey [26], golf [27], track and field [28], handball [29]. Participants involved in these interventions demonstrated greater skill acquisition, as well as greater retention of the skill. Addressing a football specific context, differential learning training has been applied successfully to football passing, football ball control, and football kicking performance [19, 30]. For example, a previous study revealed how players that enrolled in a differential learning program over a period of 4-weeks exhibited higher accuracy scores in a kicking task compared to a more traditional approach training program [30]. Also, Santos and collaborators [20] demonstrated that a 5 months training intervention sustained in differential learning embedded in small-sided games nurtured creative behaviours and favoured tactical regularity in under 13 and 15 football players. More recently, Coutinho and collaborators [21] applied a differential training program in youth football forwards at two age groups (under-15 and under-17), and it was concluded that the training program was effective to improve the overall players’ performance, mainly in the under-15 age group. Despite the proven benefits of differential learning training applied to football specific skills, there is still a gap in understanding the dose-response related to this type of approach. That is, previous studies in football have shown important improvements in players performance after enrolling in training programs that lasted from 1 to 5-months sustained on movement variability. However, to our knowledge, no study to date have addressed how the players may acutely be affected by this approach compared to more traditional approaches. Taking into consideration that the technical development is one of the major aims of coaches from youth players [31], a better understanding of the acute effects of differential learning on the players kicking performance may help coaches to better schedule and design training tasks to improve this technical action. Additionally, the effects of differential learning have been tested on technical and tactical parameters of soccer, and a natural extension of this type of work would be to determine whether or not any physical benefit can be attributed to differential learning training. In this regard, a previous study has suggested that differential learning may improve players’ strength [32]. In addition, a exploratory study also shown improvements in the players vertical jump following a differential training intervention on the squat movement [33]. Accordingly, one of the possible reasons for this increase may be linked with a higher brain activation following differential learning exercises [32], which may increase the neural drive and consequently led to better physical performances [34]. Under this perspective, it may be possible that players’ improve their physical performance while performing differential exercises to improve their kicking accuracy, however this assumption has not been tested. Based on the previous considerations, this study aimed to identify the acute effects of a differential learning intervention on football kicking performance (velocity, ball impact and accuracy) and countermovement jump (CMJ) performance in under-15 association football players. Additionally, this study also aimed to analyse how these effects were modified after a 35-min simulated eleven-a-side football match.

## Methods

### Participants

The study included 20 under-15 (U15) Portuguese football players with at least two years of football-specific training experience 1(age 13.8 ± 0.6 years, body mass 55.1 ± 11.5 kg, height 169.0 ± 8.4 cm, and body fat 9.3 ± 2.9%; goalkeepers n = 3; central defenders n = 5; fullbacks n = 3; midfielders n = 5; forwards n = 4; left-foot n = 4; right-foot n = 16). The players typically trained 4 times per week (90 to 105 minutes) and played an official game during the weekend at a regional playing standard with a duration of 70 minutes. The experimental sessions took place throughout the season on their normally scheduled training days, substituting the regularly scheduled training session. Club administrators, coaches, players and parents were fully informed of the aims and procedures of the study and signed an informed consent form to participate. All participants were notified that they could withdraw from the study at any time. The study protocol was approved and followed the guidelines stated by the Ethics Committee of the of University of Trás-os-Montes and Alto Douro, based ate Vila Real (Portugal) and conformed to the recommendations of the Declaration of Helsinki.

### Procedures

**Fig 1:**
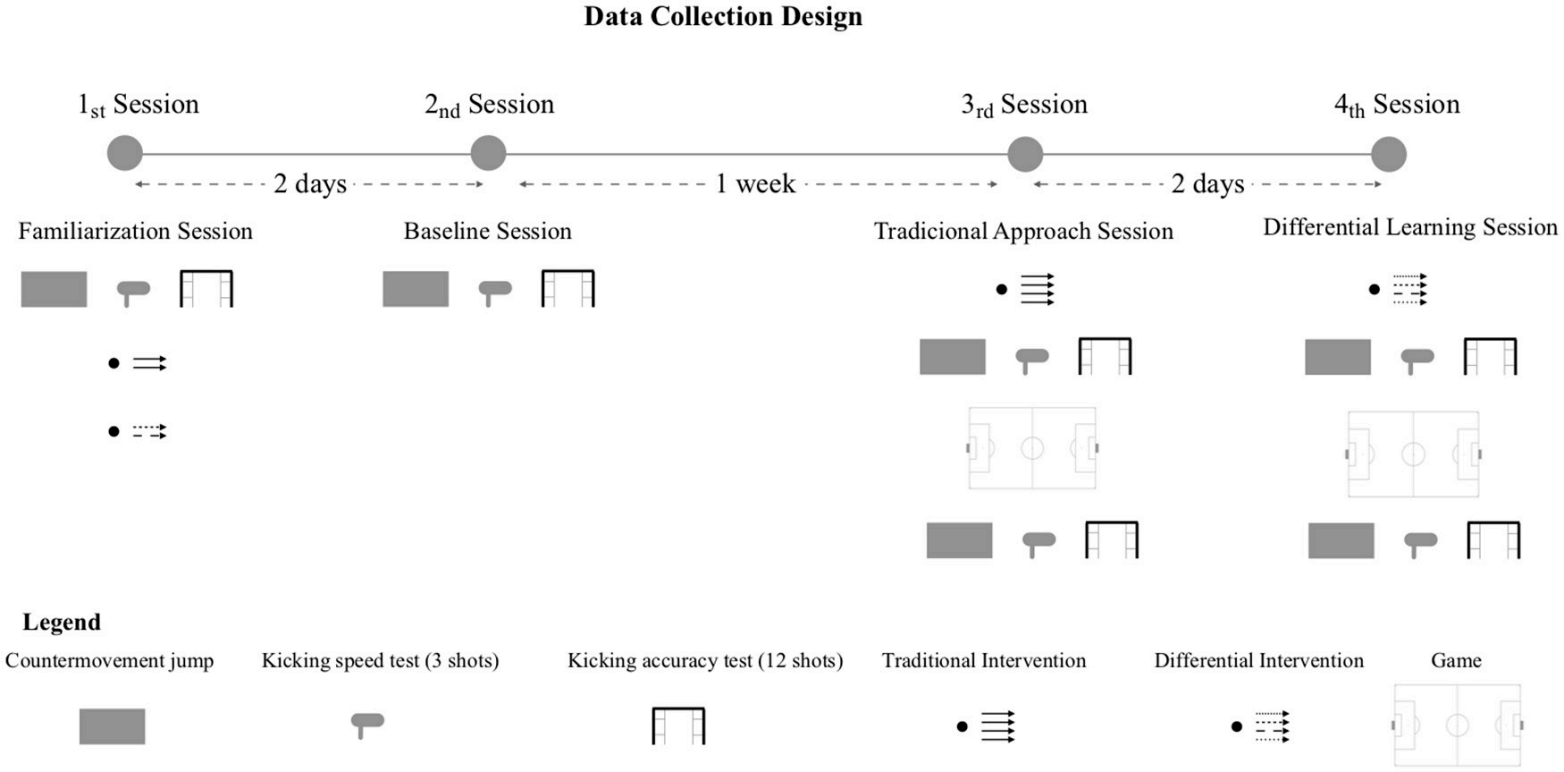
Representation of data collection design.

One familiarization session and three testing sessions were used to assess the player’s performance (Fig 1). The first and second session were performed in the first week while the third and fourth were performed on the second week to avoid possible accumulative fatiguing effects. Considering that the team had 4 training sessions per week, the sessions performed in this study were developed during the second and fourth session of each week. In addition, coaches were instructed to decrease the load of their training tasks during the remaining days.

Thus, the first served to familiarize the participants of the testing protocol and its measurements. Also, players were familiarized with the kicking actions used in both the traditional and differential interventions (18 kicks of each). The second session was used as baseline in which the players completed the pre-test battery in the following order: CMJ performance, kicking speed task, and kicking accuracy task. The third and fourth sessions were used to test the acute effectiveness of the training interventions. In these two last sessions, the players performed the training interventions, followed by the same tests used in the pre-test, administered in the same sequence. In order to assess the effects of 35-minute 11-a-side football match in the variables under study, the participants repeated the testing protocol. This period of time was selected to infer which training protocol may have longer effects, and therefore, understand which approach may be used as a warm-up strategy prior to competitive matches. Each session began with a standardized 10-minute warm up that focused predominately on low intensity running, dynamic mobility and ball possession drills, structured as the following: 2-minutes jogging, 1x30m skipping, 2x30m side run; dynamic mobility– 2x10 reps of hip adduction, 2x10 reps of hip abduction; 10 butt kicks, 10 knee raises, 10 straight leg march, 10 lateral step (5 each side); and 3 bouts of 1-minute of ball possession of 5vs5 (2 spaces) with 20x30m. Each of the testing sessions were separated by two days and all occurred at the same time and on the same training field. The players performed the training tasks wearing regular training equipment (artificial turf boots, socks, shorts and t-shirt). Although the food and water plan was not recorded, during the familiarization session, the players were instructed to follow a normal daily food and water intake [35].

### Training interventions

#### Traditional training protocol

**Fig 2:**
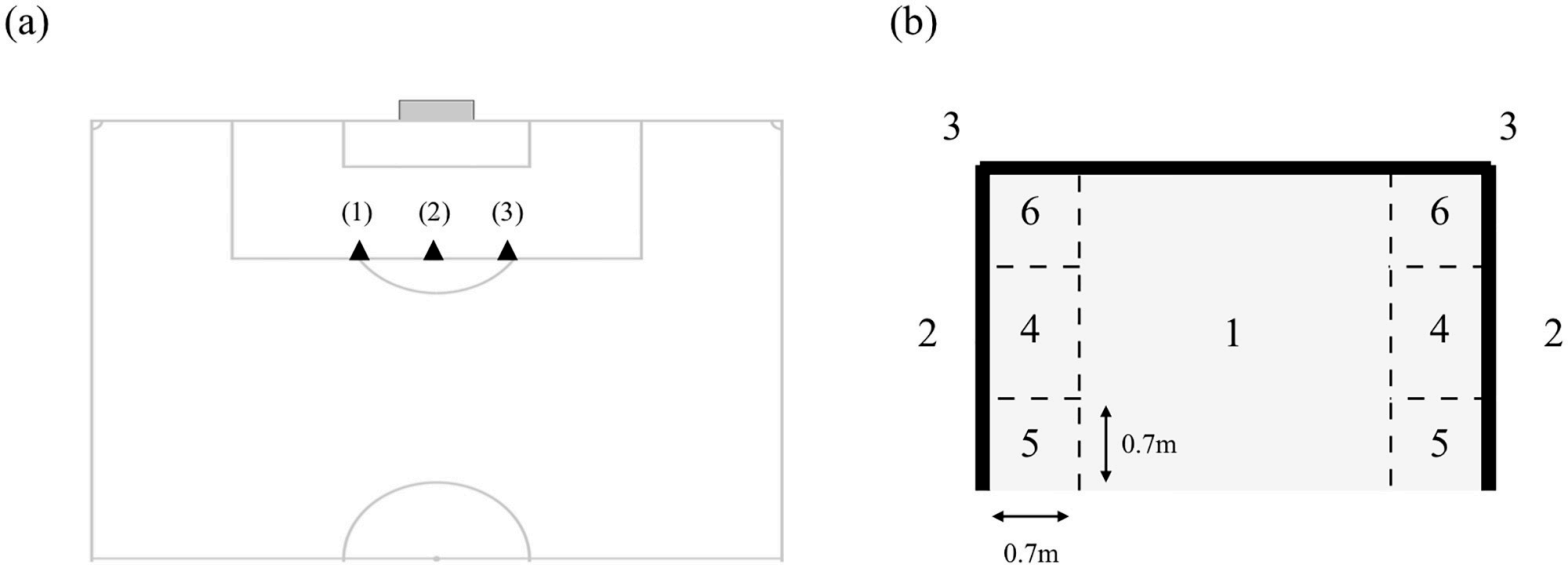
Representation of kicking accuracy task: a) field locations used in the kicking accuracy task; b) illustration of the scoring system used in the kicking accuracy task. The football goal was divided into 7 scoring zones. A score of 1 was attributed to a ball entering through the centre of the goal. A score of 2 was allotted to ball striking the frame of the goal (post & cross bar). A score of 3 was given to a ball striking the junction between post and cross bar. A score of 4 was given to a ball entering through a middle section of the goal. Scores of 5 and 6 were attributed to balls entering the bottom and top corners of the goal, respectively. Scoring areas were divided using a 0.7 m distance from the top and bottom of the goals, as well as a 0.7 m distance from the from each corner of the goal.

After completing the warm-up, the participants performed the traditional training intervention task which consisted of a total of 36 kicking repetitions in a blocked order, taken from 3 locations marked along the penalty area of the field, while using 2 different approach variations (Fig 2). The 36 kicks directed toward the goal were completed in the following sequence:

1. Six static balls were kicked toward the goal after an initial 5-meter run up from position 1.
2. Six balls were kicked toward the goal after an initial 5-meter approach from position 1.
3. Six static balls were kicked toward the goal after an initial 5-meter run up from position 2.
4. Six balls were kicked toward the goal after an initial 5-meters dribble from position 2.
5. Six static balls were kicked toward the goal after an initial 5-meter run up from position 3.
6. Six balls were kicked toward the goal after an initial 5-meter dribble from position 3.

In all repetitions, the participants were encouraged to kick the ball toward the goal with maximal accuracy and speed. Additionally, participants were provided with correctional feedback after each repetition, to ensure compliance with a biomechanically optimal movement pattern. Instructions were given by the researchers to concentrate movement feedback on one of three categories: a) error description, b) movement-oriented correction, and c) metaphoric instructions (Schöllhorn et al., 2006). Upon completing the traditional training intervention, the participants performed the post-test measurements.

#### Differential learning training protocol

During the differential learning training, participants were required to perform football kicking repetitions, using differential learning variations, in a blocked order. Participants were instructed to kick the ball in unconventional ways to increase their individual ability to adapt to the new movement patterns. In this intervention protocol, participants were not provided correctional feedback after the execution of each repetition. The execution of this intervention mirrored that of the traditional intervention. Participants were provided with the same 10-minute standardized warm up and executed 36 repetitions from the same 3 kicking locations. There were 18 different kicking variations that were designed for the intervention, and each variation was completed from kicking a static ball and after a 5-meter dribble, in order to achieve a total of 36 repetitions (see Table 1). After completing the training plan used in the differential learning intervention, the participants performed their respective post-test measurements.

**Table 1:** Example of the variations performed in differential learning intervention.

Repetition	Location	Approach (5m)	Differential Learning Variation
1	1	Run	Visual occlusion with eye patch
2	1	Run	Both arms up
3	1	Run	Rotating arms forward
4	1	Run	Hands on hips
5	1	Run	Both arms out to the side
6	1	Run	Both arms down to the side
7	2	Dribble	Arms crossed
8	2	Dribble	Both hands behind head
9	2	Dribble	Visual occlusion and right hand on hip
10	2	Dribble	Both arms extended forward
11	2	Dribble	Hands on hips and rotating hips
12	2	Dribble	Hands behind back
13	3	Run	Clapping forward and backward
14	3	Run	Left arm up and right arm out
15	3	Run	Arms down to the side and rotating hips
16	3	Run	Kick football with toe
17	3	Run	Hopping on one leg
18	3	Run	Arms extended back
19	1	Dribble	Arms crossed
20	1	Dribble	Both hands behind head
21	1	Dribble	Visual occlusion and right hand on hip
22	1	Dribble	Both arms extended forward
23	1	Dribble	Hands on hips and rotating hips
24	1	Dribble	Hands behind back
25	2	Run	Clapping forward and backward
26	2	Run	Left arm up and right arm out
27	2	Run	Arms down to the side and rotating hips
28	2	Run	Kick football with toe
29	2	Run	Hopping on one leg
30	2	Run	Arms extended back
31	3	Dribble	Visual occlusion provoked using eye patch
32	3	Dribble	Both arms up
33	3	Dribble	Rotating arms forward
34	3	Dribble	Hands on hips
35	3	Dribble	Both arms out to the side
36	3	Dribble	Both arms down to the side

### Post-match test

Upon completing both intervention protocols (traditional and differential) and the respective post-test measurements, the participants were divided into two balanced teams, according to the coach’s subjective assessment of his players physical, technical and tactical skills [36]. Then, they played a 35-minute simulated football match (Gk+10vs10+Gk) on an artificial turf pitch measuring 104×64m (length × width). Participants played according to their usual playing positions and were subjected to the same rules as an official match, without having access to their coach’s intervention. The aim of this task was to create a game-like environment that corresponded to the physical and technical demands of a regular football match. This game duration was selected as it corresponds to the length of one half of an official game for this particular population. Immediately after completing each training intervention and after completing the football match, participants were instructed to report their subjective ratings of fatigue (RPE), using a 10 point scale devised by Borg [37]. This scale has been considered as valid approach to measure exercise intensity and load [38–40]. Afterwards, the participants completed the post-match measurements in the same order as the pre-and post-test.

### Data collection

#### Countermovement jump

The players completed the CMJ in accordance with the guidelines established by Bosco, Luhtanen and Komi [41]. Prior to recording the results, participants were able to complete a maximum of 3 practice repetitions, which the researchers observed and used to correct any improper movement patterns. Participants were instructed to squat to approximately 90 degrees of knee flexion before maximally propelling themselves vertically, and to keep their hands firmly placed on their hips in order to mitigate the contribution of arm movement to the overall jump result. This task was tested using a Bosco Ergojump System (Globus Inc., Treviso, Italy) in an outdoor setting, adjacent to their regular training field. The participants were instructed to complete 2 trials interspersed by 120 seconds of rest time between attempts, and the best jump height (cm) result was documented [42].

#### Kicking task—Ball impact and speed

In the kicking task, participants were verbally instructed to use their dominant limb to execute a maximal effort kick at an official sized football goal (2.44 meters tall and 7.32 meters across) [43]. The ball was placed in a static position, 11-meters away from the centre of the goal, and each participant began the kicking approach 5-meters away from the ball. This ball position was consistent in all the kicking tests performed. The ball speed values obtained (km/h) represent the peak ball velocity immediately after impact (impact velocity) by the participants kicking foot and the average velocity of the balls trajectory from its static position to the goal (average velocity). The ball used was an official sized 5 match ball, which corresponds to what the players use in their routine training sessions and games. All of the balls used during the testing period were the same brand, size, and contained the same air pressure. Ball velocity was captured using a Doppler radar gun (Stalker Pro, Stalker Sports Radar, Plano, TX, USA; Ka band: 34.2–35.2 GHz), located in line with the initial ball position. This device has been shown to provide a high reliability of the performance of maximum kicking actions [43]. The radar gun was located centrally, 2-meters behind the frame of the goal, and held in line with ball height during the kick execution [44]. Each participant performed 3 maximal kicks, with 60 seconds allotted between each repetition. The highest release velocity of the 3 attempts was recorded, based on the methodological precedent exhibited in previous investigations measuring the velocity of a ball in a sporting context [45].

#### Kicking task accuracy

In the kicking accuracy task, participants were instructed to kick a football with their dominant foot into a goal that was divided into seven zones, each one corresponding to a level of difficulty of execution (Fig 2b). For example, balls that entered the goal in the top corners were attributed a greater value than those entering the centre of the goal. Prior to beginning the task, all participants were informed about the scoring system and were instructed to achieve as high a score as possible, in order to encourage them to complete the task without any restraint. All of the kicks were given a value from 1 through 6, with 72 being the highest achievable result. This kicking task was adapted from Schöllhorn, Hegen and Davids’ [30] research, who also assessed the effects of differential learning on football kicking accuracy. However, a few modifications/adaptations were made, including a reduction in the number of attempts from 35 to 12 and replacing the score of 1 attributed to kicks that came within 0.7 m of the outside of the goal posts by 2. In this task, each participant was required to shoot a ball at a regulation-sized football goal, without a goalkeeper, from 3 different positions (Fig 2a), and with two different approach variations. In each position, the participants were instructed to kick a ball from a static position, and again after dribbling the ball from a 5-meter distance. In total, the task involved 12 attempts completed in the following sequence:

1. Two static balls were kicked toward the goal after a 5-meter run up from position 1.
2. Two balls were kicked toward the goal after a 5-meter dribble from position 1.
3. Two static balls were kicked toward the goal after a 5-meter run up from position 2.
4. Two balls were kicked toward the goal after a 5-meter dribble from position 2.
5. Two static balls were kicked toward the goal after a 5-meter run up from position 3.
6. Two balls were kicked toward the goal after a 5-meter dribble from position 3.

After completing the aforementioned test battery above, the participants’ baseline values were established (pre-test measurements) to be compared against the scores obtained after finishing an acute session of traditional training and differential learning training.

### Statistical analysis

Individual and mean changes from pre- to post-test, as well as from baseline to post-match comparisons between the traditional and differential training were graphically represented and the variation from considered moments expressed in percentage variation (mean±SD). To realize the possibly positive (higher to differential learning)/negative (higher to traditional) effects of training interventions on players’ performance measures, the data was analysed with a specific spreadsheet for pre-post crossover trial [46]. The physical performance effects were estimated in percent units through log-transformation (to reduce the non-uniformity of error) and uncertainty in the estimate was expressed as 90% confidence limits, while the accuracy results were presented as absolute raw values. The outcome for performance measures was evaluated with the non-clinical version of magnitude-based inference. Smallest worthwhile differences were measured using the standardized units multiplied by 0.2. Uncertainty in the true effects of the conditions was assessed based on non-clinical magnitude-based inferences. Probabilities were reported using the following scale: *“<0*.*5%*, *most unlikely; 0*.*5–5%*, *very unlikely; 5–25%*, *unlikely; 25–75%*, *possibly; 75–95%*, *likely; 95–99*.*5%*, *very likely; >99*.*5%*, *most likely* [47]. Standardized (Cohen) mean differences, and respective 90% confidence intervals (CI) were also computed as magnitude of observed effect, and, thresholds were 0.2 = trivial, 0.6 = small, 1.2 = moderate, 2.0 = large, and >2.0 = very large [48].

## Results

The values of the subjective rating of perceived exertion reported after the game were similar between training approaches (traditional intervention, 4.35±0.88; differential learning intervention, 4.45±0.99).

**Fig 3:**
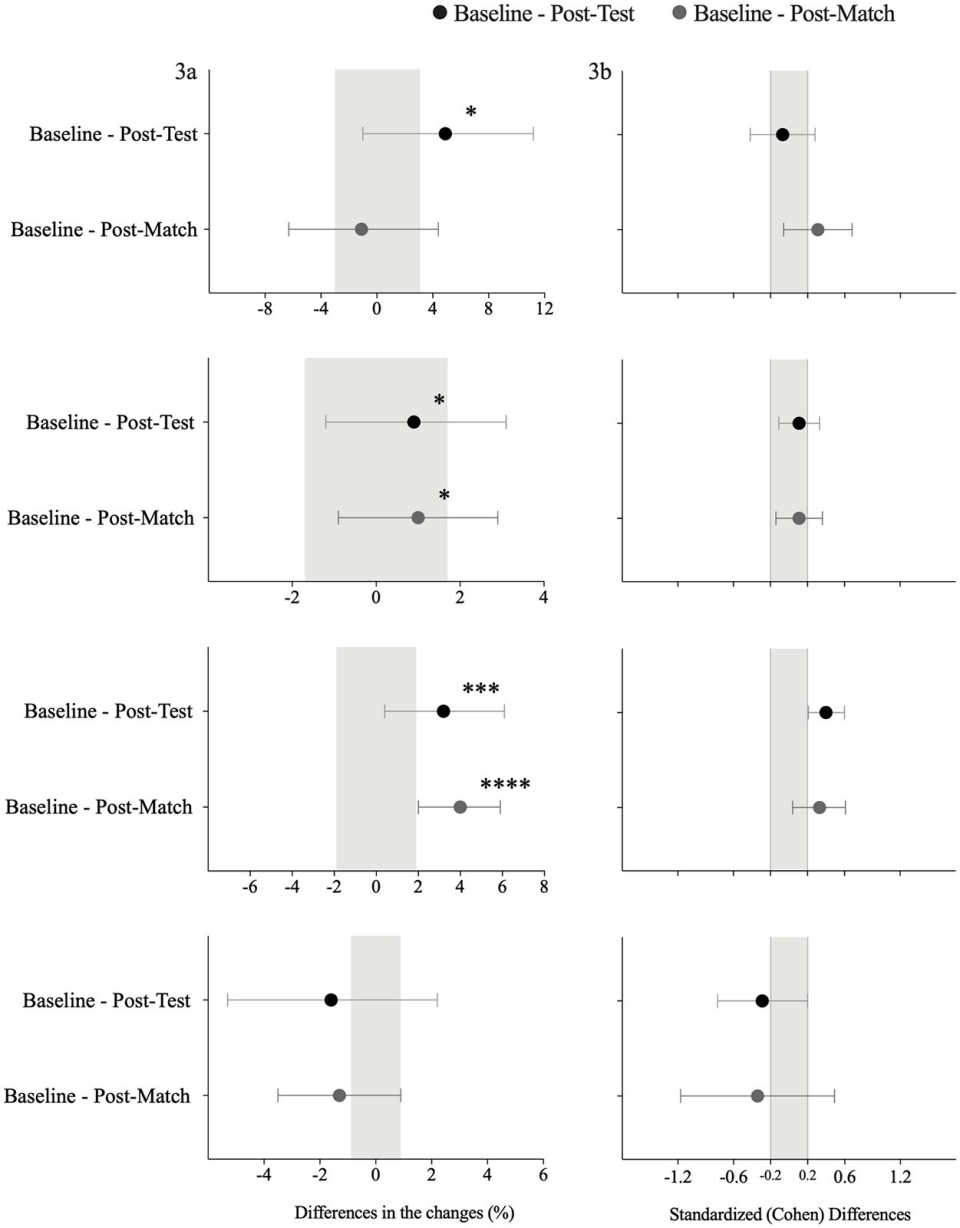
Effects of the traditional intervention (a) and differential learning intervention; (b) on CMJ (i), Ball Impact (ii), Average velocity (iii) and Accuracy (iv). Gray solid lines indicate individual responses, while black dotted lines indicated group mean value. Also, standardized (Cohen) differences; (c) of variables between the traditional and differential learning groups (black * mark represents the comparison between baseline and post-test, while the grey * mark represents the comparison between baseline and post-match test). Error bars indicate uncertainty in the true mean changes with 90% confidence intervals. Abbreviation: CMJ = countermovement jump.

The comparisons result between the traditional and differential learning approaches from the baseline to the post-test and from the baseline to the post-match are displayed in Table 2 and Fig 3. In regards to the baseline and post-test comparisons, the results of the CMJ measurements revealed that the differential learning training promoted a small increase (difference in means, %; ±90% confidence limits: 4.9%; ±6.1%, possibly) when compared with the traditional training approach. In addition, while the impact velocity expressed during the kicking task showed a trivial effect, in turn, there was a small increase in the average velocity (3.2%, ±2.8%, likely) when considering the differential learning training intervention. When assessing the differences between the results of the baseline test and the post-match measurements, an unclear trend for the accuracy and CMJ was demonstrated. Nevertheless, the differential learning approach showed a small increase (4.0%, ± 1.9%, very likely) for the average velocity when compared with the traditional approach.

**Table 2:** Descriptive statistics for the traditional vs differential learning training sessions.

Variables	Baseline–Post-test		Baseline–Post-Match	
	Difference in means; ±90% CL	Chances(negative/trivial/positive)	Difference in means; ±90% CL	Chances (negative/trivial/positive)
CMJ (cm)	4.9; ± 6.1 ↑^c^	1/29/70	-1.1; ± 5.3 ^a^	67/23/10
Impact Velocity (Km/h)	0.9; ± 2.2 ↑^c^	3/69/28	1.0; ± 1.9 ↑^c^	1/73/26
Average Velocity (Km/h)	3.2; ± 2.8 ↑^e^	0/21/78	4.0; ± 1.9 ↑^f^	0/4/96

Note: CL = confidence limits; probabilistic terms: (a) unclear; (b) unlikely; (c) possibly; (d) likely trivial; (e) likely; (f) very likely; ↓ = decrease; ↑ = increase.

**Fig 4:**
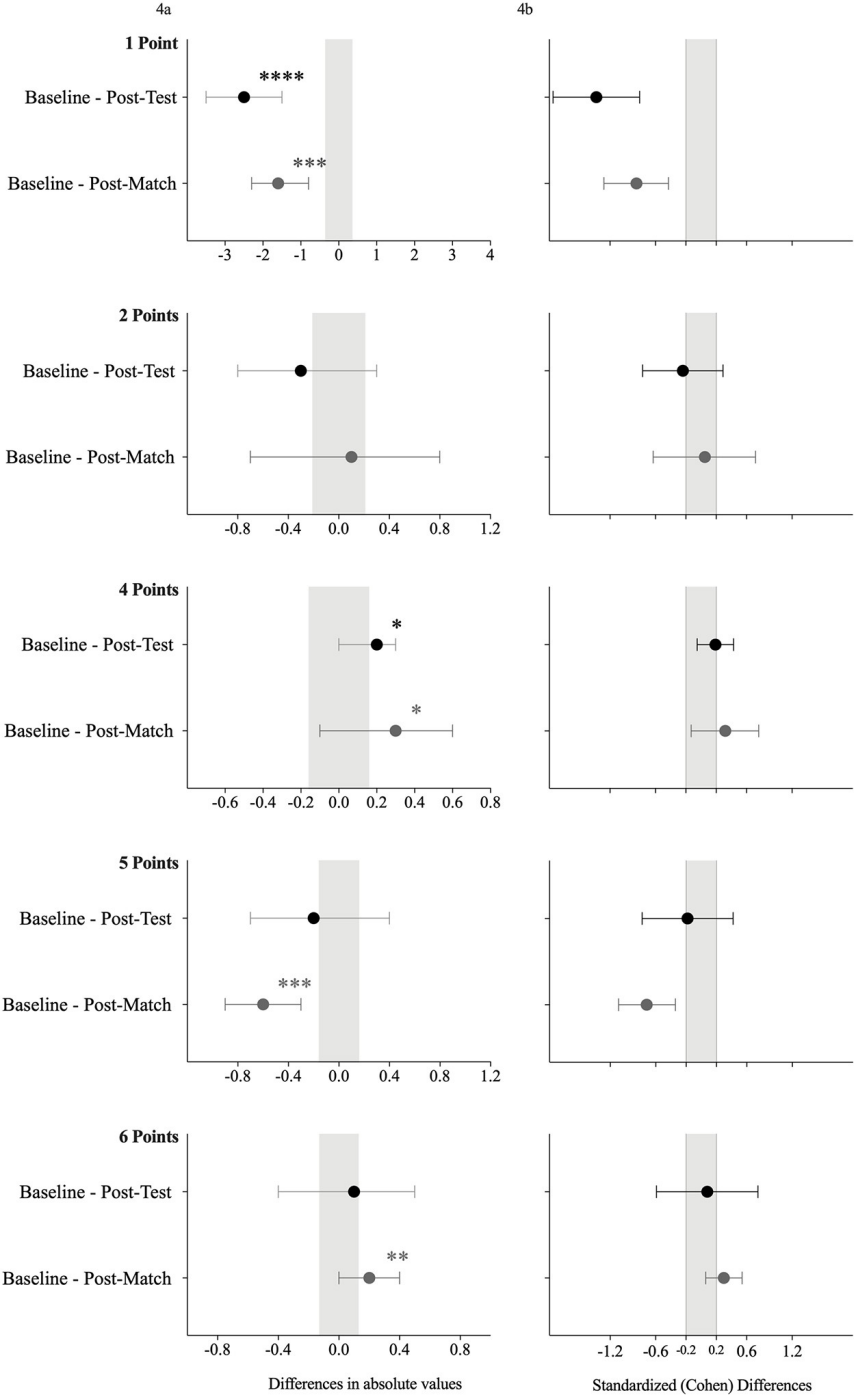
Effects of the traditional intervention and differential learning intervention on the Accuracy according to each zone (a). Gray solid lines indicate individual responses, while black dotted lines indicated group mean value. Also, standardized (Cohen) differences; (b) of variables between the traditional and differential learning groups (black mark represents the comparison between baseline and post-test, while the grey mark represents the comparison between baseline and post-match test). Error bars indicate uncertainty in the true mean changes with 90% confidence intervals. * possibly; ** likely; *** very likely; **** most likely.

The differences between the traditional and differential learning approaches for the accuracy task are presented in Table 3, Figs 3 and 4. From the baseline to the post-test, unclear effects were found between the differential learning intervention and the traditional intervention. However, individual zone analysis revealed that following the differential intervention there was a moderate decrease in accurate kicks into zone 1 (difference in means, ±90 confidence limits: -2.5, ±1.0, most likely) and a small increase in accurate kicks into zone 4 (0.2, ±0.2, possibly) compared to the traditional approach. From the baseline to the post-match comparisons, a small decrease in the overall score was found (-1.3; ±2.2, possibly) in the differential intervention compared to the traditional training intervention. From the individual zone perspective, differential learning intervention presented a moderate decrease in accurate kicks into zones 1 and 5 (-1.6; ±0.8, very likely; and -0.6± 0.3, very likely, respectively) compared to the traditional intervention. In contrast, differential learning intervention revealed a small increase in accurate kicks into zones 4 and 6 (0.3; ±0.4, possibly; and 0.2; ±0.2, likely, respectively) compared to the traditional approach.

**Table 3:** Descriptive statistics for the comparison between the traditional and differential learning interventions on the accuracy scores.

Accuracy (Zones)	Baseline–Post-test			Baseline–Post-Match		
	Difference in means; 90% CL	Chances (negative/trivial/positive)	Standardized (Cohen) Differences	Difference in means; ±90 CL	Chances (negative/trivial/positive)	Standardized (Cohen) Differences
1	-2.5; ± 1.0 ↓^h^	100/0/0	-1.4; ±0.6	-1.6; ± 0.8↓^f^	99/1/0	-0.9; ±0.4
2	-0.3; ± 0.6↓^a^	55/37/8	-0.2; ±0.5	0.1; ± 0.7 ↑^a^	27/38/35	0.1; ±0.7
3	no occurrences					
4	0.2; ± 0.2 ↑^c^	1/51/48	0.2; ±0.2	0.3; ± 0.4 ↑^c^	3/29/68	0.3; ±0.4
5	-0.2; ± 0.5 ↓^a^	48/37/15	-0.2; ±0.6	-0.6; ± 0.3 ↓^f^	99/1/0	-0.7; ±0.4
6	0.1; ± 0.4 ↑^a^	25/37/38	0.1; ±0.7	0.2; ± 0.2 ↑^e^	0/23/77	0.3; ±0.2
Total	-1.6; ± 3.8 ↓^a^	67/20/13	-0.3; ±0.8	-1.3; ± 2.2 ↓^c^	59/34/7	-0.3; ±0.5

Note: CL = confidence limits; probabilistic terms: (a) unclear; (b) unlikely; (c) possibly; (d) likely trivial; (e) likely; (f) very likely; (h) most likely; ↓ = decrease; ↑ = increase.

## Discussion

The aim of this study was to compare the acute effects of differential learning and traditional training interventions in youth football players during kicking performance and countermovement jump performance. Further, it was also inspected how these effects were modified after a 35-min simulated 11-a-side football match. In contrast to most interventions that have implemented this nonlinear pedagogical approach to teaching sport skills, this study was concerned with determining whether or not an acute performance benefit can be derived from differential learning training. With regards to the results obtained, the differential learning training increased the jumping performance and kicking velocity measures when compared with the traditional training approach, immediately after the training intervention. Additionally, kicking velocity also showed a slight increase following the post-match in the differential learning training. The results of this intervention are in alignment with Schöllhorn and collaborators [19, 30] findings, since it provides support for the acute superiority of differential learning training over more traditional training approaches in a football specific context.

It is well documented that performance in a CMJ task is strongly related to enhanced sport speed [49] and lower limb strength [50] in athletes. The better results of the CMJ test in the differential learning approach may have resulted from greater neural activation which may have impacted the recruitment of motor units and consequently lead to higher physical performance. In fact, a previous report showed higher neural activation during a differential training intervention than during a traditional approach [24]. In addition, previous studies using random practice and blocked practice found that the random group outperformed its blocker counterpart, and also exhibited greater neural activity in sensorimotor and premotor brain areas [51]. Thus, it seems possible that differential learning training provided a neurological boost to the system which resulted in elevated jumping performance. In turn, the increase in CMJ performance may have also contributed to the higher speed witnessed in kicking performance [14].

The velocity obtained during a football kick seems to be a direct consequence of the technique employed, precision and lower limb strength [6–8]. As so, the increase in the values found in the differential learning approach in both the post-test and post-match may be linked with the possible increases in neural drive, as well as more efficient movement adaptability [19]. In fact, a previous study assessing the kinematics of a handball throw after completion of a differential learning training protocol reported an increase in the players thrown ball velocity. This was related with changes in proximal-to-distal movement sequences and an alteration of individual movement pattern [29]. While these findings are promising, and present a plausible explanation for the findings exhibited in this study regarding kicking velocity, only a thorough kinetic/kinematic analysis under these proposed conditions may elucidate such claims. Hence, this study provides evidence to suggest that the acute effects of differential learning training are capable of elevating football kicking performance and that this benefit will remain throughout the duration of a competitive setting.

Previous reports have shown better kicking and passing performances in association football players’ after differential learning interventions compared to more traditional approaches [19, 30]. Different results were found in this study, since the overall accuracy (total values) showed lower mean values in the differential intervention (Δ = ~1 less from the baseline to post-test; and Δ = ~2 less from the baseline to the post-match) than in the traditional intervention. Accordingly, previous reports have shown that variable practice conditions reveal detrimental effects in short-term performances, however higher benefits can be obtained after a longer learning period [52]. However, a different trend emerged when accounting for the kicking scoring zones. While higher number of accurate kicks for zone 1 and 5 were found in the traditional intervention, in turn, the differential learning intervention showed a higher number of accurate kicks in zones 4 and 6. In this sense, the players opted to kick into zones of greater technical execution after the differential training intervention One main reason for these results may be related with different type of technical adjustment after each kick in each intervention. That is, in the differential learning intervention, the players were challenged to explore different movement patterns, without corrective feedback, which may favour their predisposition to attempt to kick into more complex zones. Furthermore, players seem to benefit from persistent adjustments to environmental constraints and are forced to engage in a self-monitoring assessment of their own performance in the differential learning approach [25], and thus, may be more able to accurately shoot into the zones of greater technical execution, such as 4 and 6. In turn, the traditional intervention received corrective feedback after each kick, which might have constrained the players execution, and possibly may have resulted in players choosing easier options.

Whilst this study showed important and insightful findings regarding the acute effects of the differential learning approach, some limitations should be acknowledged. Firstly, the tests should resemble the dynamic nature of its performance, so forthcoming training programs should analyse the acute effects of differential learning training intervention using game based approaches. In addition, the task used to analyse the players kicking accuracy allowed them to freely decide to where to kick, and therefore, a better understanding of the effects of the different training programs might have emerged if the players had only one specific zone were to kick, allowing to better compare the results. In this regard, it would be interesting if future studies analysed the time required for each player to adapt to each training intervention, as well as, which factors may have trigger the distinct learning rates in both interventions (e.g., previous sport experiences). Also, while the aim of including the simulated 35-minutes football match was to understand which approach would maintain the players performance level for a longer period, for example, to possibly included it during warm-up routines, in turn the load of this task was only controlled with the R.P.E. Although the R.P.E. have been considered as valid tool to measure exercise load, additional measurements (e.g., blood lactate) would provide a deeper understanding on the match physical demands, to understand if players’ physical exertion was similar between conditions. Admittedly, the lack of a control group in the research design debilitates the findings and refrain from achieving stronger inferences, and thus, future studies should take into consideration this limitation. Apart from these limitations, the information derived from this study certainly has applications that can be useful to athletes and coaches. Firstly, findings demonstrated that the implementation of differential learning training procedures in a team’s practice schedule slightly increased their motor output (CMJ) and kicking speed. The post-match values may also suggest that differential learning can be introduced during warm-up or pre-game routines of players as its effects withstand durations that are experienced in their competitive playing scenarios. In addition, the higher accuracy in the 4 and 6 scoring zone following the differential learning intervention may suggest this approach be recommended to increase player’s ability to kick into zones that are more difficult for the goalkeeper to defend, possibly as result of a better ability to monitor and adjust their technical performance after each kick.

## Conclusion

One of the unique aspects of this study is the contribution to understanding how differential learning training acutely alters physical and technical parameters of football kicking performance. In summary, this study demonstrated that an acute differential learning intervention was superior than a traditional training protocol with respect to CMJ performance and football kicking velocity. These results may suggest the use of a differential learning approach in the beginning of a training session since it seems to increase player’s physical performance in jumping and kicking actions. This finding was reinforced by the higher values in the kicking speed following the post-match task, indicating an important long-lasting effect. Furthermore, while the average values showed better accuracy performances following the traditional intervention, in turn, the individual zone analysis revealed that after the differential learning intervention, there were more kicks into higher zones that corresponded to a higher score. Thus, coaches could use this approach to induce movement variability and adaptability, while increasing the kicking accuracy related with zones of higher technical execution. Upon assessing the theoretical constructs of differential learning and evaluating its practical results, there is further evidence to bolster the claim that differential learning training can be incorporated into the regular training schedules of athletes, and specifically, in association football players.

## References

[1] Tierney PJ, Young A, Clarke ND, Duncan MJ. Match play demands of 11 versus 11 professional football using Global Positioning System tracking: Variations across common playing formations. Human Movement Science. 2016;49:1–8. 10.1016/j.humov.2016.05.007 27269201

[2] Russell M, Rees G, Kingsley M. Technical Demands of Soccer Match Play in the English Championship. The Journal of Strength & Conditioning Research. 2013;27(10):2869–73. 23287830 10.1519/JSC.0b013e318280cc13

[3] Bradley PS, Carling C, Gomez Diaz A, Hood P, Barnes C, Ade J, et al Match performance and physical capacity of players in the top three competitive standards of English professional soccer. Human Movement Science. 2013;32(4):808–21. 10.1016/j.humov.2013.06.002 23978417

[4] Castellano J, Casamichana D, Lago C. The Use of Match Statistics that Discriminate Between Successful and Unsuccessful Soccer Teams. Journal of Human Kinetics 2012 p. 137. 10.2478/v10078-012-0015-7 PMC3588662 23487020

[5] Torreblanca-Martinez V, Otero-Saborido F, Gonzalez-Jurado J. Effects of Muscle Fatigue Induced by Countermovement Jumps on Efficacy Parameters of Instep Ball Kicking in Soccer. Journal of applied biomechanics. 2017;33(2):105–11. 10.1123/jab.2016-0040 27735221

[6] Lees A, Nolan L. The biomechanics of soccer: a review. Journal of sports sciences. 1998;16(3):211–34. 10.1080/026404198366740 9596356

[7] Kellis E, Katis A. Biomechanical Characteristics and Determinants of Instep Soccer Kick. Journal of Sports Science & Medicine. 2007;6(2):154–65. PubMed 24149324 PMC3786235

[8] De Witt J, Hinrichs R. Mechanical factors associated with the development of high ball velocity during an instep soccer kick. Sports Biomechanics. 2012;11(3):382–90. 10.1080/14763141.2012.661757 23072048

[9] Katis A, Giannadakis E, Kannas T, Amiridis I, Kellis E, Lees A. Mechanisms that influence accuracy of the soccer kick. Journal of Electromyography and Kinesiology. 2013;23(1):125–31. 10.1016/j.jelekin.2012.08.020 23021602

[10] Manolopoulos E, Katis A, Manolopoulos K, Kalapotharakos V, Kellis E. Effects of a 10-Week Resistance Exercise Program on Soccer Kick Biomechanics and Muscle Strength. The Journal of Strength & Conditioning Research. 2013;27(12):3391–401. 23539080 10.1519/JSC.0b013e3182915f21

[11] García-Pinillos F, Martínez-Amat A, Hita-Contreras F, Martínez-López E, Latorre-Román P. Effects of a Contrast Training Program Without External Load on Vertical Jump, Kicking Speed, Sprint, and Agility of Young Soccer Players. The Journal of Strength & Conditioning Research. 2014;28(9):2452–60. 24626140 10.1519/JSC.0000000000000452

[12] Sáez de Villarreal E, Suarez-Arrones L, Requena B, Haff G, Ferrete C. Effects of Plyometric and Sprint Training on Physical and Technical Skill Performance in Adolescent Soccer Players. The Journal of Strength & Conditioning Research. 2015;29(7):1894–903. 25635606 10.1519/JSC.0000000000000838

[13] Akbulut T, Agopyan A. Effects of an Eight-Week Proprioceptive Neuromuscular Facilitation Stretching Program on Kicking Speed and Range of Motion in Young Male Soccer Players. The Journal of Strength & Conditioning Research. 2015;29(12):3412–23. 26020709 10.1519/JSC.0000000000001015

[14] Manolopoulos E, Papadopoulos C, Kellis E. Effects of combined strength and kick coordination training on soccer kick biomechanics in amateur players. Scandinavian journal of medicine & science in sports. 2006;16(2):102–10. 16533348 10.1111/j.1600-0838.2005.00447.x

[15] Schmidt R. A schema theory of discrete motor skill learning. Psychological review. 1975;82(4):225.

[16] Wulf G, Schmidt R. Variability of practice and implicit motor learning. Journal of Experimental Psychology: Learning, Memory, and Cognition. 1997;23(4):987.

[17] Schöllhorn W. Individualität-ein vernachlässigter Parameter. Leistungssport; 1999.

[18] Savelsbergh G, Kamper W, Rabius J, De Koning J, Schöllhorn W. A new method to learn to start in speed skating: A differencial learning approach. International Journal of Sport Psychology. 2010;41(4):415.

[19] Schollhorn W, Beckmann H, Michelbrink M, Sechelmann M, Trockel M, Davids K. Does noise provide a basis for the unification of motor learning theories? International journal of sport psychology. 2006;37(2/3):186.

[20] Santos S, Coutinho D, Gonçalves B, Schöllhorn W, Sampaio J, Leite N. Differential Learning as a Key Training Approach to Improve Creative and Tactical Behavior in Soccer. Research quarterly for exercise and sport. 2018:1–14. 29351500 10.1080/02701367.2017.1412063

[21] Coutinho D, Santos S, Gonçalves B, Travassos B, Wong DP, Schöllhorn W, et al The effects of an enrichment training program for youth football attackers. PLOS ONE. 2018;13(6):e0199008 10.1371/journal.pone.0199008 29897985 PMC5999098

[22] Schollhorn W, Beckmann H, Janssen D, Drepper J. 6 Stochastic perturbations in athletics field events enhance skill acquisition2010.

[23] Santos S, Memmert D, Sampaio J, Leite N. The spawns of creative behavior in team sports: A creativity developmental framework. Frontiers in psychology. 2016;7. 10.3389/fpsyg.2016.01282 PMC4999444 27617000

[24] Henz D, Schöllhorn W. Differential Training Facilitates Early Consolidation in Motor Learning. Frontiers in Behavioral Neuroscience. 2016;10. 10.3389/fnbeh.2016.00199 PMC5073148 27818627

[25] Santos S, Jiménez S, Sampaio J, Leite N. Effects of the Skills4Genius sports-based training program in creative behavior. PLOS One. 2017;12(2). 10.1371/journal.pone.0172520 28231260 PMC5322953

[26] Beckmann H, Winkel C, Schöllhorn W. Optimal range of variation in hockey technique training. International Journal of Sport Psychology. 2010;41(4):5–45.

[27] Porter J, Magill R. Systematically increasing contextual interference is beneficial for learning sport skills. Journal of sports sciences. 2010;28(12):1277–85. 10.1080/02640414.2010.502946 20845219

[28] Schöllhorn W, Beckmann H, Janseen D, Drepper J. Stochastic perturbation in athletics field events enhance skill acquisition Motor learning in practice–A constraints-led approach. London Routledge 2010.

[29] Wagner H, Muller E. The effects of differential and variable training on the quality parameters of a handball throw. Sports Biomechanics. 2008;7(1):54–71. 10.1080/14763140701689822 18341136

[30] Schollhorn W, Hegen P, Davids K. The nonlinear nature of learning-A differential learning approach. The Open Sports Sciences Journal. 2012;5(1).

[31] Lloyd RS, Cronin JB, Faigenbaum AD, Haff GG, Howard R, Kraemer WJ, et al National Strength and Conditioning Association Position Statement on Long-Term Athletic Development. Journal of strength and conditioning research / National Strength & Conditioning Association. 30(6). 10.1519/JSC.0000000000001387 26933920

[32] Torrents C, Balagué N, of and … P-J. Linear and nonlinear analysis of the traditional and differential strength training. Baltic Journal of Sport and …. 2007.

[33] Hegen P, Polywka G, Schöllhorn WI. The differential Learning Approach in Strength Training (Squat). In: Radmann A, Hedenborg S, Tsolakidis E, editors. Book of Abstract of 20th annual Congress of the European College of Sport Science. Malmö2015. p. 590.

[34] Ranganathan VK, Siemionow V, Liu JZ, Sahgal V, Yue GH. From mental power to muscle power—gaining strength by using the mind. Neuropsychologia. 2004;42(7):944–56. Epub 2004/03/05. 10.1016/j.neuropsychologia.2003.11.018 14998709

[35] Owen AL, del WP, McKenna M, Dellal A. Heart rate responses and technical comparison between small- vs. large-sided games in elite professional soccer. J Strength Cond Res. 25(8). Epub 2011/06/07. 10.1519/JSC.0b013e3181f0a8a3 21642858

[36] Coutinho D, Goncalves B, Wong DP, Travassos B, Coutts AJ, Sampaio J. Exploring the effects of mental and muscular fatigue in soccer players’ performance. Hum Mov Sci. 2018;58:287–96. Epub 2018/03/20. 10.1016/j.humov.2018.03.004 29549745

[37] Borg G. Psychophysical bases of perceived exertion. Medicine & Science in Sports & Exercise. 1982;14(5):377–81. 7154893

[38] Impellizzeri FM, Rampinini E, Coutts AJ, Sassi A, Marcora SM. Use of RPE-based training load in soccer. Med Sci Sports Exerc. 2004;36(6):1042–7. Epub 2004/06/05. 10.1249/01.mss.0000128199.23901.2f . 15179175

[39] Naidu SA, Fanchini M, Cox A, Smeaton J, Hopkins WG, Serpiello FR. Validity of Session Rating of Perceived Exertion Assessed via the CR100 Scale to Track Internal Load in Elite Youth Football Players. Int J Sports Physiol Perform. 2019;14(3):403–6. Epub 2018/09/12. 10.1123/ijspp.2018-0432 . 30204528

[40] Rodriguez-Marroyo JA, Antonan C. Validity of the session rating of perceived exertion for monitoring exercise demands in youth soccer players. Int J Sports Physiol Perform. 2015;10(3):404–7. Epub 2014/09/10. 10.1123/ijspp.2014-0058 . 25202917

[41] Bosco C, Luhtanen P, Komi PV. A simple method for measurement of mechanical power in jumping. European journal of applied physiology and occupational physiology. 1983;50(2):273–82. Epub 1983/01/01. 10.1007/bf00422166 . 6681758

[42] Thomas K, French D, Hayes P. The effect of two plyometric training techniques on muscular power and agility in youth soccer players. The Journal of Strength & Conditioning Research. 2009;23(1):332–5. 19002073 10.1519/JSC.0b013e318183a01a

[43] Markovic G, Dizdar D, Jaric S. Evaluation of tests of maximum kicking performance. Journal of sports medicine and physical fitness. 2006;46(2):215 16823350

[44] Sterzing T, Hennig E. The influence of soccer shoes on kicking velocity in full-instep kicks. Exercise and Sport Sciences Reviews. 2008;36(2):91–7. 10.1097/JES.0b013e318168ece7 18362691

[45] Sterzing T, Lange J, Wächtler T, Müller C, Milani T, editors. Velocity and accuracy as performance criteria for three different soccer kicking techniques. ISBS-Conference Proceedings Archive; 2009.

[46] Hopkins W. Spreadsheets for Analysis of Controlled Trials, with Adjustment for a Subject Characteristic 2006 [2016]. http://sportsci.org/2006/wghcontrial.htm.

[47] Hopkins WG, Marshall SW, Batterham AM, Hanin J. Progressive Statistics for Studies in Sports Medicine and Exercise Science. Med Sci Sport Exer. 2009;41(1):3–12. 10.1249/MSS.0b013e31818cb278 19092709

[48] Batterham A, Hopkins W. Making meaningful inferences about magnitudes. International journal of sports physiology and performance. 2006;1(1):50–7. 19114737

[49] Cronin J, Hansen K. Strength and power predictors of sports speed. J Strength Cond Res. 2005;19(2):349–57. 10.1519/14323.1 . 15903374

[50] Nuzzo J, McBride J, Cormie P, McCaulley G. Relationship between countermovement jump performance and multijoint isometric and dynamic tests of strength. J Strength Cond Res. 2008;22(3):699–707. 10.1519/JSC.0b013e31816d5eda . 18438251

[51] Cross E, Schmitt P, Grafton S. Neural substrates of contextual interference during motor learning support a model of active preparation. J Cogn Neurosci. 2007;19(11):1854–71. 10.1162/jocn.2007.19.11.1854 . 17958488

[52] Williams M, Hodges N. Practice, instruction and skill acquisition in soccer: Challenging tradition. Journal of Sports Sciences. 2005;23(6):637–50. 10.1080/02640410400021328 16195012

